# Assessment of the Concentrations of Selected Aminothiols in Patients after COVID-19

**DOI:** 10.3390/jcm13144108

**Published:** 2024-07-14

**Authors:** Izabela Szołtysek-Bołdys, Wioleta Zielińska-Danch, Danuta Łoboda, Krzysztof S. Gołba, Beata Sarecka-Hujar

**Affiliations:** 1 Department of General and Inorganic Chemistry, Faculty of Pharmaceutical Sciences in Sosnowiec, Medical University of Silesia in Katowice, 41-200 Sosnowiec, Poland; iboldys@sum.edu.pl (I.S.-B.); wzdanch@sum.edu.pl (W.Z.-D.); 2 Department of Electrocardiology and Heart Failure, Medical University of Silesia in Katowice, 40-635 Katowice, Poland; dana.loboda@gmail.com (D.Ł.); kgolba@sum.edu.pl (K.S.G.); 3 Department of Electrocardiology, Upper-Silesian Medical Centre in Katowice, 40-635 Katowice, Poland; 4 Department of Basic Biomedical Science, Faculty of Pharmaceutical Sciences in Sosnowiec, Medical University of Silesia in Katowice, 41-200 Sosnowiec, Poland

**Keywords:** COVID-19, aminothiols, homocysteine, cysteine, glutathione, cardiovascular diseases

## Abstract

**Background**: Data show that due to endothelial damage and thrombogenic effects, severe acute respiratory syndrome coronavirus-2 (SARS-CoV-2) infection may accelerate the development of atherosclerosis and increase the risk of cardiovascular diseases (CVDs). The impaired metabolism of aminothiols increases oxidative stress, as these molecules are involved in antioxidant defense as well as in thiol redox control. In this study, total levels of selected aminothiols (i.e., cysteine (Cys), homocysteine (HCy), and glutathione) in convalescents after coronavirus disease of 2019 (COVID-19) were evaluated. The analyses were made according to the sex of the patients, time from COVID-19 onset, and COVID-19 severity. **Methods**: The study group consisted of 212 patients after COVID-19. Levels of total aminothiols were assessed in the blood plasma using high-performance liquid chromatography (HPLC). **Results**: The mean Cys concentrations were higher in men than in women (229.92 µmol/L ± 51.54 vs. 210.35 µmol/L ± 41.90, respectively; *p* = 0.003). Differences in Cys levels were also noticed in the total study group between patients distinguished due to time from disease onset (226.82 µmol/L ± 40.57 in <12 weeks, 232.23 µmol/L ± 47.99 in patients 12–24 weeks, and 208.08 µmol/L ± 48.43 in patients >24 weeks; *p* = 0.005). In addition, over 11% of total patients 12–24 weeks from disease onset had Cys levels above 300 µmol/L compared to almost 4% of patients <12 weeks and 2% of patients >24 weeks (*p* = 0.046). In sex-adjusted subgroups, significant differences due to time from COVID-19 were found in Cys levels in women (*p* = 0.004) and in glutathione levels in men (*p* = 0.024). None of the aminothiol levels differed between the subgroups based on the severity of COVID-19. **Conclusions**: Men had overall higher Cys levels than women. Cys levels were lower >24 weeks after COVID-19 onset than in the earlier period after disease onset. A partial elevation in Cys levels 12–24 weeks after the disease onset may contribute to the increase in CVD risk in the post-COVID-19 period.

## 1. Introduction

The SARS-CoV-2 pandemic, which occurred between 2019 and 2023, remains a severe challenge to the health system. Cardiovascular complications related to COVID-19 occurred both in the acute phase of infection and a few weeks after recovery, including, among others, acute coronary syndromes, Takotsubo syndrome, myocarditis, cardiac arrhythmias, thromboembolic complications, exacerbation of heart failure, new-onset hypertension, postural orthostatic tachycardia syndrome, and stroke [1,2,3,4,5,6,7]. Mechanisms that contribute to cardiovascular complications during and after SARS-CoV-2 infection include angiotensin-converting enzyme 2, the renin-angiotensin-aldosterone system, and the kinin-kallikrein system [8]. Moreover, promoting systemic inflammation, endothelial dysfunction, and arterial stiffness, COVID-19 can also accelerate the development of atherosclerosis and increase the risk of atherosclerotic cardiovascular diseases (ASCVDs) [9,10,11,12]. The question arises of whether SARS-CoV-2 infection increases the ASCVD risk by increasing non-classical cardiovascular risk factors.

Low-molecular-weight aminothiols like Cys, HCy, and glutathione have been suggested as non-classical markers of various disease states in the body, including ASCVD [13,14]. Both Cys and HCy are amino acids derived from methionine. Cys is a precursor of glutathione and is structurally similar to HCy. HCy damages the endothelium and intima through increased proliferation of vascular smooth muscle cells, decreased nitric oxide synthesis, and overproduction of oxidative radicals [15,16]. In turn, thiolactone, a highly reactive product of HCy oxidation, modulates the expression of genes that encode proteins essential in vascular homeostasis pathways and promotes atherogenesis [17]. Elevated levels of HCy (i.e., levels above 15 µmol/L) are considered toxic to cells and thus may be associated with the etiology of various health problems [18,19]. In a Taiwanese study [20], a higher ASCVD risk (calculated by the Framingham cardiovascular risk score) was found in middle-aged and elderly patients who had elevated HCy levels. This study estimated HCy as an independent risk factor for ASCVD [20]. Concentrations of HCy, particularly in correlation with D-dimer, have also been positively associated with venous and pulmonary thromboembolism [21,22]. Conflicting results appear in the literature regarding Cys levels. Only some studies indicate an association of high Cys levels with coronary artery disease (CAD) [23,24]. However, El-Khairy et al. [25] reported the highest risk for ASCVD when low tCys and high tHcy levels coexisted. In addition, a previous study by Lima et al. [26] demonstrated significantly higher concentrations of cysteine in plasma and a higher frequency of patients with hypercysteinemia (i.e., levels above 300 µmol/L) in a group with CAD than in controls. No such correlations were found for HCy, which allowed the authors to conclude that Cys is a better predictor of CAD than HCy [26]. As for glutathione level, Musthafa et al. [27] demonstrated a reduced level of erythrocyte glutathione and an increased oxidative status in patients with premature CAD compared to age-matched controls. Similarly, a recent study based on CAD patients from India also reported significantly lowered glutathione levels in patients compared to controls [28].

It is known that due to endothelial damage and thrombogenic effects, SARS-CoV-2 infection may accelerate the development of atherosclerosis and increase the risk of ASCVD [9,10,29]. Moreover, in some patients with severe COVID-19, high HCy and low glutathione levels have been observed and used as a biomarker to determine COVID-19 severity and lung complications [30,31]. HCy levels in patients during and after COVID-19 were shown to be related to thrombogenic complications [32]. A study by Tu et al. [33] in a group of men under 50 years of age with stroke after SARS-CoV-2 infection showed that the HCy concentration was increased in 22% of the subjects, with a significantly high level of HCy at 93 μmol/L in one of the patients. In diabetic patients, elevated levels of inflammatory markers such as C-reactive protein and HCy three months after COVID-19 indicate prolonged inflammation of the arteries and an increased risk of thrombosis, indicating a persistently high risk of cardiovascular complications [34]. Recent studies indicate that in infected cells, the SARS-CoV-2 virus affects folate and one-carbon metabolism to meet the demands of viral replication [35,36]. It is hypothesized that elevated HCy and redox imbalance may be associated with chronic fatigue and cognitive impairment in long-COVID syndrome [35,36,37].

In the present study, our goal was to evaluate total levels of selected aminothiols, i.e., Cys, HCy, and glutathione, as non-classical risk factors for cardiovascular disease in convalescents hospitalized as part of a comprehensive cardiopulmonary rehabilitation program after COVID-19. We analyzed these parameters regarding the sex, time from disease onset, and COVID-19 severity.

## 2. Materials and Methods

### 2.1. Study Groups

The study was retrospective and cross-sectional. The study group consisted of adult patients in the convalescence phase after COVID-19 who, under the National Health Fund (NHF) program, participated in inpatient cardiopulmonary rehabilitation at the Cardiac Rehabilitation Department of the Ustroń Health Resort (Poland) for up to 12 months from the initial diagnosis. The rehabilitation inclusion criteria were complications of symptomatic SARS-CoV-2 infection in the respiratory, cardiovascular, nervous, or musculoskeletal system; decrease in muscle strength; and persistent dyspnea of the intensity of 2–3 on the modified Medical Research Council dyspnea scale. An experienced pulmonologist conducted the recruitment process. Active COVID-19 was excluded in all patients based on qualitative tests for the presence of the SARS-CoV-2 antigen in nasopharyngeal swabs. The exact inclusion and exclusion criteria were described previously [38]. Of the 553 consecutive patients participating in the rehabilitation NHF program in 2021–2022, 231 subjects agreed to have blood samples taken for testing. Eventually, considering the various difficulties in obtaining blood samples (hemolysis and the inability to collect), 212 adequate samples for laboratory tests of selected aminothiols were provided.

Based on the interview and diagnoses from the medical records, a description of the comorbidities and the course of the acute period of COVID-19 (with the severity of the disease classified as mild, moderate, severe, and critical by the guidelines of the Polish Society of Epidemiologists and Infectiologists [39]) were obtained. In the study, due to the very low number of patients with a critical severity of COVID-19, we analyzed severe and critical patients jointly (severe/critical), and at the same time, we also combined the mild and moderate groups into one group (mild/moderate).

Since we did not recruit a typical control group of healthy people for this study, and the concentration of aminothiols may be significantly elevated in patients with ASCVD, we compared a cohort of patients after COVID-19 with a group of ASCVD patients recruited in parallel (*n* = 95, average age 65.08 years ± 15.79). These patients were hospitalized in the Department of Electrocardiology, Upper-Silesian Medical Center in Katowice (Poland) in 2022–2023. This reference group was specifically selected as the patients did not exhibit symptoms suggesting COVID-19 or positive test results for SARS-CoV-2 over the preceding 12 months.

The study protocol complied with the ethical guidelines of the 1975 Declaration of Helsinki and was approved by the Bioethical Committee (no. of approval: PCN/CBN/0052/KB1/68/I/21/22). Each recruited individual provided written informed consent.

### 2.2. Definitions Used to Classify Co-Existing Conditions

A patient was included in the hypertensive group if they had a systolic blood pressure (SBP) ≥ 140 mmHg or DBP ≥ 90 mmHg or if the patient was taking antihypertensive drugs. Hypercholesterolemia was diagnosed when the total cholesterol (TC) was ≥190 mg/dL, the low-density lipoprotein cholesterol (LDL) was ≥115 mg/dL, or the participant was on lipid-lowering medications. Chronic kidney disease was diagnosed when creatinine clearance calculated from the Cockcroft-Gault formula was <60 mL/min. Smoking status was assessed by questionnaire and patients were divided into smokers who currently smoke cigarettes, former smokers who had not smoked cigarettes for at least one month (in our study, the time since quitting smoking in people classified as former smokers was measured in years (mean 18.99 years ± 12.82)), and nonsmokers who had never smoked cigarettes.

### 2.3. Biochemical Analyses of Aminothiols

The concentrations of the biochemical parameters were obtained from the blood plasma. Biological samples of antecubital venous blood were collected after 12 h of fasting, and the samples were centrifuged within two hours of being drawn.

The Cys, HCy, and glutathione concentrations were established using the high-performance liquid chromatography (HPLC) method, according to the previously described methodology [40]. In addition, the ratio of Cys/HCy was calculated for each patient.

### 2.4. Statistical Analyses

Statistical analysis was performed using STATISTICA 13.0 software (STATSOFT; Statistica, Tulsa, OK, USA). The continuous variables were presented as mean values (M) and the standard deviations (SD), while the categorical variables were shown as the absolute numbers (n) and the relative numbers (%). In the current study, a total of 212 COVID-19 convalescents were studied. Considering the large size of the sample, it can be assumed that the variable follows the normal distribution, and due to the central limit theorem, it can be thought that the distribution of these data converges to a normal distribution. The quantitative data between the two subgroups were compared using Student’s *t*-test. For the comparison of the continuous variables between more than two groups of patients, ANOVA tests were used. In the case of significance, a post hoc analysis was performed. The Pearson correlation coefficient was computed to assess the relationships between HCy and Cys concentrations. The stochastic independence Chi^2^ test was used to compare the categorical variables between subgroups. If the expected number of cases was below 5, a comparison of categorical data between groups was made with the Fisher exact test. To analyze polytomous categorical variables between subgroups depending on the time from COVID-19 onset and severity of the disease (i.e., 2 × 3), the Freeman–Halton extension of the Fisher exact test was used (VassarStats, Poughkeepsie, NY USA). When the *p*-value was below 0.05, the result was considered statistically significant.

During the study, a power analysis was performed based on differences in selected continuous variables (i.e., level of cysteine, fasting glucose, and HDL level) and categorical variables (i.e., frequencies of hypertension and CAD) between men and women using a two-tailed test with a significance level of 0.05. For continuous parameters, we obtained over 80% of the power while for categorical variables the power was 70%. After discussion, we found this power acceptable. The sample size of 212 COVID-19 convalescents was satisfactory for us considering the study was performed in a single medical center.

## 3. Results

### 3.1. General Characteristics of the Study Group

The characteristics of the study group after COVID-19 and sex subgroups are shown in Table 1. Age and BMI did not differ between women and men. However, both sex subgroups differed in terms of smoking status; significantly greater numbers of former and active smokers were demonstrated in the male subgroup than in the female subgroup. Men had significantly lower HDL levels than women. Conversely, male patients after COVID-19 had substantially higher TG and fasting glucose levels than women (Table 1).

Among the comorbidities, men after COVID-19 suffered from CAD and hypertension significantly more often than women (Table 1). Other chronic diseases did not differentiate the studied subgroups.

### 3.2. Levels of Analyzed Aminothiols in Sex Subgroups of Patients after COVID-19

In Table 2, mean levels of the selected aminothiols between women and men after COVID-19 are presented. Levels of Cys differ significantly between sex subgroups. For mean concentrations of glutathione, and the mean Cys/HCy ratio, no differences were demonstrated. In turn, the mean HCy levels were higher in men compared to women; however, the difference was close to significance.

In the study group, we did not find a correlation between HCy levels and age of the patients.

**Figure 1:**
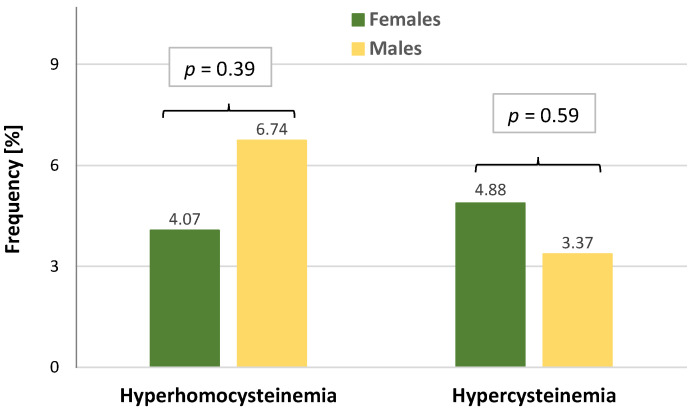
The frequencies of hyperhomocysteinemia and hypercysteinemia in sex subgroups of patients with COVID-19.

The frequencies of individuals with hyperhomocysteinemia (i.e., a level of HCy above 15 μmol/L) were comparable between females and males. Similarly, no difference was found for the prevalence of individuals with hypercysteinemia (i.e., with Cys levels above 300 μmol/L) (Figure 1).

### 3.3. Levels of Analyzed Aminothiols in the Subgroups of Post-COVID-19 Patients

The levels of analyzed aminothiols were compared between the subgroups of post-COVID-19 patients depending on the time from the disease onset and the severity of the acute disease. Table 3 shows the results of the comparisons between the subgroups of patients based on the time from the disease onset. Significant differences between the subgroups depending on time from disease onset were found for mean Cys levels. A slight increase in Cys concentration was observed in the period of 12–24 weeks from COVID-19 onset. Then, the mean concentrations of Cys were significantly decreased in patients suffering from COVID-19 for more than 24 weeks before recruitment to the study compared to patients 12–24 weeks from COVID-19 onset (*p* = 0.005). The frequency of patients with hypercysteinemia also differed between the distinguished subgroups (*p* = 0.046). The highest percentage of patients with Cys level above 300 µmol/L was observed within the subgroup 12–24 weeks from disease onset. In the case of HCy levels, the highest mean level of HCy was observed in patients <12 weeks from the disease onset while the lowest was in patients >24 weeks from COVID-19 onset, but the difference was on the bound of significance (*p* = 0.06).

**Figure 2:**
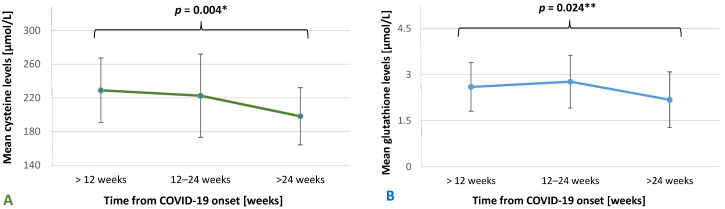
Mean (M) Cys and glutathione levels depending on time from the COVID-19 onset in sex subgroups. (**A**)—female subgroup; * post hoc analysis: <12 vs. >24, *p* = 0.018 and 12–24 vs. >24, *p* = 0.031; (**B**)—male subgroup; ** post hoc analysis: 12–24 vs. >24, *p* = 0.021. The standard deviation (SD) bars are presented in the chart. Significant differences are in bold.

In the sex subgroup analysis depending on time from COVID-19 onset, we observed significant differences in Cys levels in women and glutathione levels in men (Figure 2A,B). The mean levels of Cys were comparable between women who were <12 weeks and 12–24 weeks from COVID-19 onset (229.05 µmol/L ± 38.33 vs. 222.58 µmol/L ± 49.34, respectively; *p* = 0.54). The lowest mean level of Cys was in women >24 weeks from the disease onset (198.24 µmol/L ± 33.99) and this value was significantly lower than the mean Cys in women both <12 weeks and 12–24 weeks (*p* = 0.018 and *p* = 0.031, respectively). In the men subgroup, patients 12–24 weeks had the highest mean Cys levels compared to men < 12 weeks and men > 24 weeks, but these differences were not significant (241.62 µmol/L ± 45.36, 224.04 µmol/L ± 38.70, and 226.22 µmol/L ± 64.21, respectively; *p* = 0.31). There was also no statistical disparity in Cys levels between sexes in patients 12–24 weeks from disease onset. In addition, in men, there was a significant trend in the distribution of mean glutathione values (*p* = 0.021). The mean levels of glutathione were comparable between men <12 weeks and men 12–24 weeks from COVID-19 onset (2.60 µmol/L ± 0.79 vs. 2.77 µmol/L ± 0.86, respectively; *p* = 0.85). The lowest mean level of glutathione was in men >24 weeks from the disease onset (2.18 µmol/L ± 0.91) and this value was significantly lower than the mean glutathione in men 12–24 weeks after COVID-19 (*p* = 0.021).

The results of comparisons between the subgroups of patients based on the disease severity are demonstrated in Table 4.

None of the aminothiols differentiated the subgroups based on the severity of the disease. We observed a tendency to higher levels of HCy and Cys and a higher value of Cys/HCy ratio, as well as a higher percentage of patients with hypercysteinemia in the patients with severe/critical COVID-19 compared to mild/moderate COVID-19 patients. The mean value of the Cys/HCy ratio was comparable between the groups based on the severity of the disease. In turn, the mean level of gluthathione and the percentage of patients with hypercysteinemia tended to be elevated in patients from the mild/moderate group. However, the mild/moderate group was almost four times larger than the severe/critical group.

### 3.4. Correlations between HCy and Cys Levels

The Pearson correlation coefficient between levels of Hcy and levels of Cys was assessed in the total group as well as sex subgroups. We observed a weak positive correlation between HCy concentrations and the levels of Cys in the total group of COVID-19 patients (r = 0.204, *p* = 0.003) as well as in the female subgroup (r = 0.248, *p* = 0.006). The higher the HCy level, the greater the level of Cys on average. In men, no correlation between HCy and Cys was demonstrated.

### 3.5. Comparisons of Aminothiols Levels between COVID-19 Patients and the Reference Group of Patients with ASCVD

We found that the COVID-19 group and ASCVD group did not differ in mean HCy concentration, although the HCy level was slightly higher in ASCVD patients. However, more patients with HCy levels above 15 µmol/L were found in the ASCVD group than in the COVID-19 group (Table 5). On the other hand, Cys levels were significantly lower in the CVD group, and the percentage of patients with Cys levels above 300 µmol/L was also more minor compared to COVID-19 patients, but nonsignificant. Still, the ASCVD group’s mean Cys/HCy ratio was significantly lower (Table 5). Cardiac patients also had considerably lower mean levels of glutathione compared to COVID-19 patients.

## 4. Discussion

COVID-19 is a transient CVD risk factor [41,42]. In the post-COVID period, the risk of new-onset CVD increases in the first four weeks after COVID-19 and then decreases to the usual level in the period 13–52 weeks after disease [41]. Moreover, the increased risk of atherosclerotic complications is independent of age, sex, and the main risk factors of ASCVD [42]. This post-COVID-19-associated increase in CVD risk, as well as the coexistence of symptoms of long-COVID syndrome, may result from various mechanisms. Currently, the main focus is on the chronic state of vascular endothelial dysfunction, which causes damage to multiple organs [43]. In the present group of patients after COVID-19, we assessed the levels of selected biochemical parameters which are usually analyzed in the context of cardiac risk. In a population of 23 million adults analyzed by Koyama et al. [44], all COVID-19 patients were at significantly higher risk of cardiovascular complications. The authors also observed a greater risk of CVD after the acute phase of the disease (over 30 days) among patients both with and without prevalent diabetes, which shows the need for monitoring for incident CVD beyond the first 30 days after diagnosis of COVID-19 [44].

The amionthiols we selected for analyses are non-classical risk factors for CVD. The reference values for HCy plasma concentrations are commonly considered normal when they are between 5 and 15 μmol/L [45]. However, such reference ranges are not clearly defined for Cys levels [46]. It was reported that normal ranges of Cys are between 240 and 360 μmol/L [47] although in the study by El-Khairy et al. [25], a Cys range from 250 to 275 μmol/L was considered the reference. Most often, available data include information on the concentration of HCy as a marker of atherosclerosis [13,14,15,17,18,19,20]. It is assumed that hyperhomocysteinemia is an HCy concentration above 15 µmol/L, and in particular, HCy levels in the range of 16–100 µmol/L are defined as mild hyperhomocysteinemia and HCy concentrations above 100 µmol/L are considered severe hyperhomocysteinemia [13]. In the study by Kryukov et al. [48], based on patients with COVID-19, it was assumed that hyperhomocysteinemia concerns an Hcy concentration above 10 µmol/L, and the average value of HCy was 7.9 µmol/L. The range of HCy concentrations in commercial plasma has been determined to be 5–15 µmol/L [49], and the suggested mean HCy concentration value is 7.5 µmol/L. In our study, the average HCy level was 9.50 µmol/L, which was higher than that reported in the above-mentioned studies. It should be noted the concentration of HCys we established concerned patients after COVID-19 and not in the acute phase of the disease. On the other hand, in patients analyzed before COVID-19 by Hortin et al. [50], the average concentration of HCy was 9 µmol/L and the average age of the subjects was 54 years old, which is comparable to the age of our group of patients. Recently, the HCy level was suggested as an important parameter in the follow-up of COVID-19 [51]. In a study based on Turkish patients, an association of Hcy levels with poor prognosis was observed; patients transferred from the ward to the intensive-care unit had significantly higher mean HCy levels compared to patients who did not require such intervention (7.10 µmol/L vs. 5.80 µmol/L, respectively). It was shown that each unit increase in Hcy levels increased the risk of death in COVID-19 patients almost two-fold (OR = 1.75) [52].

As for another analyzed aminothiol, we observed that the mean Cys level was significantly higher in men than in women after COVID-19. Only a few previous studies have reported differences in mean Cys concentrations between women and men [25,53,54]. Data by El-Khairy et al. [25] in a large group of 750 patients with vascular disease demonstrated statistically higher levels of Cys in men compared to women, which is consistent with our results. In this study, the average Cys concentration was 275 µmol/L, but it concerned people aged 40–50; however, the study took place well before the COVID-19 pandemic [25]. Similarly, the same Cys discrepancy between sex subgroups was observed in healthy adults [53]. On the other hand, a study by Sobczak et al. [54] demonstrated opposing Cys levels in healthy women and men depending on smoking status, i.e., in nonsmokers Cys levels were higher in women while in passive smokers and active smokers, men had higher Cys levels. The concentrations of Cys in our total COVID-19 group were comparable to the Cys levels in COVID-19 patients studied by Kryukov et al. [48] (218 µmol/L and 227 µmol/L, respectively). As for the level of glutathione, it was higher in analyzed female COVID-19 convalescents than in men, but the difference was close to the bound of significance. Regarding the distribution of cardiovascular risk factors, we did not observe any special differences between sexes, i.e., in terms of biochemical parameters, TG was significantly higher in men and HDL in women, but TC, LDL, and non-HDL levels did not differ between sexes. Moreover, in terms of comorbidities, hypertension was more common in men, which is consistent with observations on the general population [55], but percentages of diabetes mellitus, hypercholesterolemia, and venous thrombosis were comparable between sexes.

In our study, levels of Cys differed significantly between the subgroups depending on the time from disease onset. In patients that were >24 weeks after COVID-19 onset, the mean concentrations of Cys were significantly lower compared to patients 12–24 weeks from disease onset. In the female subgroup, mean levels of Cys in women >24 weeks from COVID-19 onset were significantly lower compared to women both <12 weeks and 12–24 weeks from the disease. We also observed that there was the highest percentage of patients with a Cys level above 300 µmol/L among patients that were 12–24 weeks from disease onset compared to <12 weeks and >24 weeks subgroups.

Our total analyzed group of COVID-19 patients as well as sex subgroups showed an approximately 23-fold lower Hcy concentration than Cys level. A study by Hortin et al. [50] showed similar orders of aminothiol concentrations. In turn, the mean level of glutathione was 2.60 µmol/L, while in the study by Kryukov et al. [48] the concentration of glutathione was 1.24 µmol/L. However, it should be noted that this was a group of subjects with severe COVID-19 infection. The range of glutathione concentration in commercial human plasma is between 2 and 5 µmol/L [49]. The present research demonstrated that recovering from COVID-19 infection did not reduce glutathione concentration below 2 µmol/L. Glutathione is one of the most important antioxidants that protect brain cells against the attack of free radicals. An efficient antioxidant defense system is extremely important for the proper functioning of the brain throughout the many years of human life. Glutathione concentration decreases with age and in neurotransmitter diseases [56]. Horovitz et al. [57] found that glutathione administered to patients with dyspnea secondary to COVID-19 pneumonia alleviated “cytokine storm syndrome” and alleviated respiratory distress. Recent data from Turkey [58] demonstrated that diabetic COVID-19 patients had the lowest levels of superoxide dismutase levels and total antioxidant status, while they had the highest total oxidant status and oxidative stress index compared with healthy controls.

Compared to CVD patients, COVID-19 patients had significantly higher mean Cys levels. Also, the average value of the Cys/HCy ratio was significantly greater in the COVID-19 group than in patients with CVD. Among cardiac patients, we observed a reduction in glutathione concentration below 2 µmol/L, which adversely affects the immune system of these patients. However, conducting further research in this area is currently difficult because most CVD patients have contracted COVID-19.

Our study has some limitations. The first one is a retrospective study design based partially on survey and medical record data. The second one is the inclusion criteria for the study, linked to the eligibility criteria for the post-COVID-19 Cardiac Rehabilitation program of the NHF. The third one is the lack of a healthy reference group. The group of CVD patients, despite meeting the assumptions of no SARS-CoV-19 infection, was not healthy and as such cannot serve as a typical reference group. In addition, COVID-19 patients came from different parts of Poland, and they were treated in the acute phase of COVID-19 in various medical centers and had completed the treatment process, which was a certain limitation because we could not examine how much the analyzed parameters increased at the peak of the disease or to what values they dropped. The current study also lacks an assessment of vascular damage biomarkers that could link the aminothiol-mediated oxidative stress change to endothelial dysfunction following COVID-19, which would provide valuable insights. And lastly, the study derived from a single medical center and was limited only to the patients who agreed to participate in this specific medical center.

## 5. Conclusions

The post-COVID-19 patients had significantly higher mean Cys levels and a higher average Cys/HCy ratio compared to CVD patients, with a partial elevation in Cys levels 12–24 weeks after the disease. These findings may indicate a significant increase in the atherosclerosis process and a potential increase in CVD risk among post-COVID-19 convalescents, highlighting the importance of long-term monitoring and care.

## Figures and Tables

**Table 1:** General and biochemical characteristics of the analyzed groups of patients and frequencies of comorbidities.

	Total COVID-19 Group *N* = 212	Female Subgroup *N* = 123	Male Subgroup *N* = 89	*p*-Value
**General parameters**				
Age (years), M ± SD	58.95 ± 8.22	59.42 ± 8.01	58.30 ± 8.51	0.33
BMI (kg/m^2^), M ± SD	29.28 ± 4.77	29.17 ± 5.21	29.43 ± 4.13	0.69
Smoking status, n (%)				**<0.001**
Former smokers	78 (36.79)	38 (30.89)	40 (44.94)	
Smokers	13 (6.13)	3 (2.44)	10 (11.24)	
Nonsmokers	121 (57.08)	82 (66.67)	39 (43.82)	
**Biochemical parameters**				
TC (mg/dL), M ± SD	238.12 ± 62.42	243.87 ± 59.78	230.17 ± 65.40	0.11
LDL (mg/dL), M ± SD	145.99 ± 40.47	149.76 ± 39.43	140.77 ± 41.53	0.11
HDL (mg/dL), M ± SD	69.66 ± 28.06	76.24 ± 26.72	60.56 ± 27.47	**<0.001**
TG (mg/dL), M ± SD	192.35 ± 112.71	170.29 ± 71.22	222.85 ± 147.68	**<0.001**
Non-HDL ^1^ (mg/dL), M ± SD	168.46 ± 50.58	167.63 ± 51.00	169.62 ± 50.27	0.70
Fasting glucose (mg/dL), M ± SD	92.46 ± 22.68	89.26 ± 17.67	96.88 ± 27.69	**0.015**
CRP (mg/L), M ± SD	3.62 ± 4.31	3.88 ± 5.09	3.25 ± 2.92	0.29
D-dimers, (mg/L), M ± SD	1.16 ± 7.13	1.54 ± 0.31	0.62 ± 0.73	0.35
Troponin (ng/mL), M ± SD	0.49 ± 0.30	0.46 ± 0.28	0.52 ± 0.32	0.18
**Comorbidities**				
CAD, n (%)	22 (10.38)	7 (5.69)	15 (16.85)	**0.009**
Hypertension, n (%)	127 (59.91)	65 (52.85)	62 (69.66)	**0.013**
Diabetes, n (%)	41 (19.34)	21 (17.07)	20 (22.47)	0.33
Hypercholesterolemia, n (%)	103 (48.58)	54 (43.90)	49 (55.06)	0.11
History of ischemic stroke, n (%)	1 (0.47)	1 (0.81)	0 (0.00)	0.99 *
Chronic kidney disease, n (%)	1 (0.47)	1 (0.81)	0 (0.00)	0.99 *
Venous thrombosis, n (%)	5 (2.36)	3 (2.44)	2 (2.25)	0.99 *

M—mean; SD—standard deviation; CAD—coronary artery disease; BMI—body mass index; TC—total cholesterol; LDL—low-density lipoprotein; HDL—high-density lipoprotein; TG—triglycerides; CRP—C-reactive protein. ^1^ non–HDL cholesterol was calculated by subtracting HDL from TC. *—2-tailed Fisher exact test. Significant differences are in bold.

**Table 2:** Levels of selected aminothiols (HCy, Cys, and glutathione) and Cys/HCy ratio in sex subgroups.

Aminothiols	Total COVID-19 Patients *N* = 212	Females *N* = 123	Males *N* = 89	*p*-Value
HCy (µmol/L), M ± SD	9.50 ± 3.21	9.17 ± 3.14	9.96 ± 3.26	0.08
Cys (µmol/L), M ± SD	218.56 ± 47.08	210.35 ± 41.90	229.92 ± 51.54	**0.003**
Glutathione (µmol/L), M ± SD	2.60 ± 0.91	2.69 ± 0.90	2.48 ± 0.91	0.11
Cys/HCy ratio, M ± SD	24.81 ± 8.19	24.82 ± 7.83	24.80 ± 8.70	0.98

M—mean; SD—standard deviation; Cys—cysteine; HCy—homocysteine. Significant differences are in bold.

**Table 3:** Levels of selected aminothiols (HCy, Cys, and glutathione) and Cys/HCy ratio in COVID-19 patients subgroups depending on time from disease onset.

Aminothiols	COVID-19 Patients <12 Weeks from Disease Onset N = 27	COVID-19 Patients 12–24 Weeks from Disease Onset N = 71	COVID-19 Patients >24 Weeks from Disease Onset N = 91	*p*-Value
HCy (µmol/L), M ± SD	9.95 ± 2.66	9.85 ± 2.79	9.13 ± 3.78	0.06
Cys (µmol/L), M ± SD	226.82 ± 40.57	232.23 ± 47.99	208.08 ± 48.43	**0.005 ^1^**
Glutathione (µmol/L), M ± SD	2.75 ± 0.88	2.78 ± 0.83	2.50 ± 0.95	0.11
Cys/HCy ratio, M ± SD	24.26 ± 7.56	25.60 ± 9.72	24.61 ± 7.18	0.68
Hyperhomocysteinemia, n (%)	2 (7.41)	4 (5.63)	2 (2.20)	0.28 *
Hypercysteinemia, n (%)	1 (3.70)	8 (11.27)	2 (2.20)	0.039 *

M—mean; SD—standard deviation; Cys—cysteine; HCy—homocysteine; ^1^ post hoc analysis for Cys level: patients 12–24 weeks vs. patients >24 weeks *p* = 0.005; *—the Freeman-Halton extension of the Fisher exact test; Significant differences are in bold.

**Table 4:** Levels of selected aminothiols (HCy, Cys, and glutathione) and Cys/HCy ratio in COVID-19 patients subgroups depending on the severity of acute COVID-19.

Aminothiols	Mild/Moderate *N* = 163	Severe/Critical *N* = 45	*p*-Value
HCy (µmol/L), M ± SD	9.39 ± 2.95	9.91 ± 4.08	0.64
Cys (µmol/L), M ± SD	217.73 ± 44.47	224.06 ± 56.55	0.28
Glutathione (µmol/L), M ± SD	2.65 ± 0.92	2.45 ± 0.83	0.33
Cys/HCy ratio, M ± SD	24.88 ± 8.22	24.87 ± 8.25	0.54
Hyperhomocysteinemia, n (%)	6 (3.68)	3 (6.25)	0.65
Hypercysteinemia, n (%)	9 (5.52)	2 (4.44)	0.93

M—mean; SD—standard deviation; Cys—cysteine; and HCy—homocysteine.

**Table 5:** Comparison of levels of aminothiols analyzed in the study (HCy, Cys, and glutathione) and values of Cys/HCy ratio in COVID-19 patients and the reference group of CVD patients.

Aminothiols	COVID-19 Patients *N* = 212	ASCVD Group *N* = 95	*p*-Value
HCy (µmol/L), M ± SD	9.50 ± 3.21	10.22 ± 4.81	0.79
Cys (µmol/L), M ± SD	218.56 ± 47.08	151.00 ± 55.76	**<0.001**
Glutathione (µmol/L), M ± SD	2.60 ± 0.91	1.77 ± 0.76	**<0.001**
Cys/HCy ratio, M ± SD	24.81 ± 8.19	16.75 ± 6.97	**<0.001**
Hyperhomocysteinemia, n (%)	9 (4.25)	11 (11.58)	**0.021**
Hypercysteinemia, n (%)	11 (5.19)	2 (2.11)	0.19

M—mean; SD—standard deviation; ASCVDs—atherosclerotic cardiovascular diseases; Cys—cysteine; and HCy—homocysteine. Significant differences are in bold.

## Data Availability

The data presented in this study are available on request in the Department of Basic Biomedical Science, Faculty of Pharmaceutical Sciences, Medical University of Silesia in Katowice (Poland). The data are not publicly available due to privacy restrictions.
